# Effects of Sodium-Glucose Co-Transporter 2 Inhibitors on Vascular Cell Function and Arterial Remodeling

**DOI:** 10.3390/ijms22168786

**Published:** 2021-08-16

**Authors:** William Durante, Ghazaleh Behnammanesh, Kelly J. Peyton

**Affiliations:** Department of Medical Pharmacology and Physiology, University of Missouri, Columbia, MO 65212, USA; gb3nc@mail.missouri.edu (G.B.); durantek@health.missouri.edu (K.J.P.)

**Keywords:** sodium-glucose co-transporter 2 inhibitors, diabetes, endothelial dysfunction, cardiovascular disease, neointima formation, arterial stiffness

## Abstract

Cardiovascular disease is the leading cause of morbidity and mortality in diabetes. Recent clinical studies indicate that sodium-glucose co-transporter 2 (SGLT2) inhibitors improve cardiovascular outcomes in patients with diabetes. The mechanism underlying the beneficial effect of SGLT2 inhibitors is not completely clear but may involve direct actions on vascular cells. SGLT2 inhibitors increase the bioavailability of endothelium-derived nitric oxide and thereby restore endothelium-dependent vasodilation in diabetes. In addition, SGLT2 inhibitors favorably regulate the proliferation, migration, differentiation, survival, and senescence of endothelial cells (ECs). Moreover, they exert potent antioxidant and anti-inflammatory effects in ECs. SGLT2 inhibitors also inhibit the contraction of vascular smooth muscle cells and block the proliferation and migration of these cells. Furthermore, studies demonstrate that SGLT2 inhibitors prevent postangioplasty restenosis, maladaptive remodeling of the vasculature in pulmonary arterial hypertension, the formation of abdominal aortic aneurysms, and the acceleration of arterial stiffness in diabetes. However, the role of SGLT2 in mediating the vascular actions of these drugs remains to be established as important off-target effects of SGLT2 inhibitors have been identified. Future studies distinguishing drug- versus class-specific effects may optimize the selection of specific SGLT2 inhibitors in patients with distinct cardiovascular pathologies.

## 1. Introduction

Diabetes mellitus is a metabolic disease that occurs due to a deficiency of insulin production and/or action, which is characterized by a state of chronic hyperglycemia [1]. The prevalence of diabetes, especially type 2 diabetes mellitus (T2DM), is rising rapidly in most regions of the world and represents a major global cause of premature mortality [2,3]. Cardiovascular disease is the principle driver of mortality and morbidity in diabetes. Patients with diabetes have a two- to four-fold increased rate of death due to vascular disease, resulting in a decidedly shortened life span [4,5,6]. Diabetes is strongly associated with the development of both microvascular, including retinopathy, nephropathy, neuropathy, and macrovascular complications, such as ischemic heart disease, peripheral vascular disease, and cerebrovascular disease, that results in organ and tissue damage in approximately one-third to one-half of individuals [7,8,9]. Atherosclerosis is a major contributor to the macrovascular manifestations of the disease. Patients with diabetes develop atherosclerosis at a younger age, with greater severity, and with a wider distribution relative to patients without the disease [7]. Furthermore, diabetic patients exhibit greater rates of restenosis following percutaneous coronary revascularization and stenting [10,11,12]. Besides its adverse effect on the length and the quality of life, the growing diabetes pandemic threatens to inflict an economically costly demand on health services, further highlighting the need to develop new therapies to manage the vascular complications triggered by this disease [13].

While the etiology of vascular disease in diabetes is complex and multidimensional, defects in endothelial cell (EC) and vascular smooth muscle cell (VSMC) function play an essential role. The endothelium lines the intimal surface of blood vessels and serves as a critical modulator of vascular function and structure. It dynamically controls vascular permeability and tone, inflammation, vessel wall morphology and composition, and thrombosis by generating a myriad of mediators, of which nitric oxide (NO) plays a primary role [14,15,16]. The release of NO by endothelial NO synthase (eNOS) executes a vital task in safeguarding vascular health. It modulates blood pressure and blood flow by inhibiting vascular tone. NO also elicits antithrombotic and anti-inflammatory effects by hindering the aggregation and adhesion of platelets and the recruitment, infiltration, and activation of leukocytes within the vessel wall. Furthermore, NO blocks VSMC proliferation and migration as well as oxidative phosphorylation. Disruption of NO synthesis results in the dysfunction of ECs and the loss of their homeostatic function, leading to EC apoptosis, enhanced endothelial permeability, impaired endothelium-dependent vasodilation, EC activation, inflammation, thrombosis, and neointima formation, which together aids in the development of vascular disease. Endothelial malfunction, as measured by depressed NO levels and endothelium-dependent vasodilation, is a characteristic feature of diabetes that has been detected in both humans and animals [17,18,19,20,21]. In addition, diminished proliferation and migration of ECs is frequently observed in diabetes, and this may contribute to the muted angiogenic response noted in many diabetic tissues [22,23,24]. Interestingly, several of the metabolic abnormalities observed in T2DM, including hyperglycemia, excess free fatty acid liberation, and insulin resistance, contribute to EC dysfunction by influencing the synthesis and/or degradation of NO [25]. A number of downstream effector molecules have been identified that negatively impact the bioavailability of NO in diabetes, including oxidative stress, inflammatory mediators, reduced sensitivity of the phosphatidylinositol-3 kinase (PI3K)/Akt pathway that activates eNOS, and accelerated formation of advanced glycation end products (AGEs) [26]. AGEs are protein and lipids that are glycated as a result of exposure to sugars. AGEs interact with the receptor for AGEs (RAGE) stimulating ROS production and the sustained activation of the pro-inflammatory transcription factor nuclear factor-κB (NF-κB) that drives inflammation in diabetes mellitus. 

VSMC dysfunction also contributes to vascular pathologies in diabetes. VSMCs are highly differentiated cells found within the medial layer of blood vessels [27]. They play a critical role in regulating blood pressure and blood distribution, as well as maintaining the structural integrity of the blood vessel. VSMCs express a unique constellation of proteins that are required for their contractile function. However, VSMCs possess remarkable plasticity and readily shift from a differentiated, quiescent, contractile phenotype to a synthetic phenotype that is characterized by the loss of their contractile proteins and increases in extracellular matrix synthesis, and elevated rates of proliferation and migration in response to various stimuli and environmental cues [28]. This phenotypic switching allows VSMCs to participate in vascular remodeling leading to the repair of injured blood vessels, but if not properly controlled it can promote the development of occlusive vascular disease such as postangioplasty restenosis, atherosclerosis, pulmonary arterial hypertension, and aneurysm formation [29,30,31,32]. Notably, VSMC proliferation and migration is augmented in diabetes facilitating the development of intimal lesions [33], and as a result diabetic patients account for a significant proportion of all peripheral vascular and coronary revascularization procedures [34,35]. 

Sodium-glucose cotransporter 2 (SGLT2) inhibitors are the newest class of glucose-lowering agents [36]. These drugs act by inhibiting glucose reabsorption in the proximal tubule of the kidney leading to glycosuria and decreases in both fasting and postprandial glycemia in patients with T2DM. A large number of these compounds have been developed and four have been approved for use by the Food and Drug Administration and the European Medicines Agency (canagliflozin, dapagliflozin, empagliflozin, and ertugloflozin), while others have been licensed in Japan and India (ipragliflozin, luseogliflozin, tofogliflozin, and remogliflozin). A number of large clinical trials have convincingly demonstrated that SGLT2 inhibitors improve cardiovascular outcomes in T2DM patients, including a reduced risk of cardiovascular death and hospitalization for heart failure [37,38,39,40]. These trials led to the current international recommendation that patients with T2DM and cardiovascular disease should receive a SGLT2 inhibitor along with metformin regardless of baseline or personalized glycosylated hemoglobin (HbA_1C_) levels. Intriguingly, the reduction of adverse cardiovascular events occurs rapidly following the start of therapy, suggesting that mechanisms other than improvements in hyperglycemia are responsible for this effect as glycemic control requires years to have a measureable impact [41,42]. Consistent with this notion, post hoc analysis of clinical trials indicates that baseline HbA_1C_ or reductions in HbA_1C_ are not associated with any treatment benefit with SGLT2 inhibitors [37,38,39,40]. Moreover, dapagliflozin was recently shown to be efficacious even in patients without diabetes [43], providing further proof that the salutary action of SGLT2 inhibitors extend beyond its anti-glycemic action.

Numerous hypotheses have been proposed to explain the cardiovascular benefits of SGLT2 inhibitors, including reductions in body mass, adipose tissue, blood pressure, plasma uric acid levels, plasma volume and inflammation, improvements in renal function and lipid profile, liver steatosis, cardiac structure, increases in natriuresis and hematocrit, and alterations in energy metabolism [44,45,46]. In addition, the ability of SGLT2 inhibitors to prevent atherosclerosis has been speculated to contribute to the cardioprotective action of these drugs, and this topic has been the subject of a number of excellent recent reviews [47,48,49]. In this focused review, we discuss the effects of SGLT2 inhibitors on endothelial NO synthesis, vascular cell function, and arterial remodeling, and propose that the direct actions of SGLT2 inhibitors on ECs and VSMCs contribute to their favorable effects on the cardiovascular system. 

## 2. Effect of SGLT2 Inhibitors on Endothelium-Dependent Vasodilation

The impact of SGLT2 inhibitors on EC function has been investigated in numerous clinical studies. Administration of empagliflozin for 6 months significantly improves EC function, as assessed by flow-mediated dilation (FMD), in T2DM patients with established coronary artery disease [50]. Multiple regression analysis indicates that the change in plasma triglyceride levels is the strongest predictive factor for the improvement in FMD. In addition, 12 weeks of empagliflozin treatment improves EC function in patients with type 1 diabetic mellitus (T1DM) [51]. Another, small exploratory study found that empagliflozin markedly elevates brachial artery shear stress and FMD in T2DM, and suggests that empagliflozin-mediated increases in wall shear stress, secondary to a rise in hematocrit and blood viscosity, stimulates the release of NO which underlies the beneficial effects of the drug on EC function [52]. However, a recently published multicenter, randomized, placebo-controlled, double-blind trial found that 24 weeks of empagliflozin treatment of T2DM patients is not associated with an improvement in endothelial function, as measured by reactive hyperemia peripheral tonometry [53]. Both canagliflozin and tofogliflozin have been demonstrated to improve EC function in T2DM patients with heart failure [54,55]. The enhancement in EC function by canagliflozin is immediate and consistently observed throughout a 12-month follow-up period. Dapagliflozin add-on therapy to metformin for 16 weeks also increases brachial artery FMD in patients with inadequately controlled early-stage T2DM [56]. The improvement in FMD likely involves a reduction of oxidative stress, as demonstrated by the decline in urinary 8-hydroxy-2′-deoxyguanosine. Dapagliflozin also augments peripheral microvascular endothelial function in patients with poorly controlled T2DM that corresponds to reductions in patient’s blood pressure and abdominal fat mass [57]. Furthermore, an active-controlled trial found that dapagliflozin corrects endothelial dysfunction in T2DM patients with atherosclerotic disease [58]. In contrast, a prospective, randomized, blinded end point study showed that dapagliflozin preserves renal artery vasodilating capacity in T2DM patients with hypertension, but it fails to modify FMD [59]. Thus, many, but not all, clinical studies support a role for SGLT2 inhibitors in alleviating endothelial dysfunction in diabetes. Variances between the studies may reflect differences in dose and duration of SGLT2 inhibitor treatment, patient populations, background medications, use of different techniques to measure EC function, and potential off-target effects of specific SGLT2 inhibitors, among other factors.

Preclinical studies have consistently documented a salutary effect of SGLT2 inhibitors on NO bioavailability and EC function. An early report found that empagliflozin improves eNOS phosphorylation (activation) and endothelium-dependent vasodilation in streptozotocin (STZ)-induced T1DM rats by interfering with oxidative stress and AGE-RAGE signaling [60]. Treatment with empagliflozin also restores eNOS phosphorylation and EC function while limiting AGE expression and nitrosative stress in the db/db mouse model of T2DM [61]. These beneficial effects occurred independent of any changes in blood pressure and body weight. Similarly, empagliflozin improves coronary microvascular function as reflected by the increase in coronary flow velocity reserve in obese, pre-diabetic ob/ob mice [62]. Interestingly, empagliflozin elevates the arginine/asymmetric dimethylarginine ratio in these animals, which would provide additional substrate to drive eNOS-mediated NO production. Furthermore, empagliflozin-mediated improvements in endothelium-dependent vasodilation have been noted in a co-morbid rheumatoid arthritis/T2DM rat model, Zucker diabetic fatty rats, metabolic syndrome ZSF1 rats, and in STZ-treated apolipoprotein-E (apoE)-deficient mice [63,64,65,66]. Amelioration of EC dysfunction in these studies is associated with increases in vascular eNOS activity, eNOS and AMP-activated kinase (AMPK) expression, and decreases in systolic blood pressure, vasoconstrictor eicosanoids, angiotensin-II, endothelin-1, P-selectin, vascular cell adhesion protein-1 (VCAM-1), and AGE-RAGE signaling. In addition, dapagliflozin administration improves endothelial function and significantly reduces vascular adhesion molecule expression and NF-κB activation, and macrophage vessel wall infiltration in apoE-null mice fed a high fat diet, and attenuates endothelial dysfunction in diabetic db/db mice [67,68]. The SGLT2 inhibitor ipragliflozin also restores eNOS activity and endothelium-dependent vasorelaxation in STZ-induced diabetic mice [69]. Therefore, a preponderance of evidence supports a role for SGLT2 inhibitors in preserving NO bioavailability and endothelial function in diabetes by influencing the activity or expression of numerous molecules that impact eNOS, oxidative stress, inflammation, vascular reactivity, and blood pressure. 

## 3. Effect of SGLT2 Inhibitors on EC Function 

### 3.1. SGLT2 Inhibitors Increase NO Bioavailability

SGLT2 inhibitors exert significant direct effects on ECs. In an early study, the natural occurring SGLT2 inhibitor phlorozin was found to restore NO synthesis in human umbilical vein ECs (HUVEC) treated with palmitic acid [70]. The amelioration of endothelial dysfunction is dependent on the activation of the PI3K/Akt/eNOS signaling pathway, but how phlorozin stimulates PI3K to elevate NO levels is not known. Similarly, empagliflozin and dapagliflozin prevents the tumor necrosis factor-alpha (TNFα)-mediated loss of NO bioavailability in human coronary artery ECs [71]. However, in this case, the SGLT2 inhibitors have no effect on eNOS expression, activity, or localization, but they abolish the rise in intracellular reactive oxygen species (ROS) evoked by TNFα, suggesting that SGLT2 inhibitors elevate NO levels by interfering with the scavenging of NO by ROS. Using a co-culture system of human cardiac microvascular ECs (CMECs) and rat ventricular myocytes, Juni et al. [72] demonstrated that CMECs positively affect cardiac function, principally through the release of endothelium-derived NO. Furthermore, they showed that exposure to uremic serum mitigates the endothelium-mediated improvements in cardiomyocyte contractility, and that empagliflozin counters the detrimental action of uremic serum on CMECs. Mechanistically, uremic serum stimulates the production of mitochondrial ROS that accumulate in the cytoplasm and limit the bioavailability of NO. By blocking the generation of mitochondrial oxidants, empagliflozin retards the scavenging of NO, independent of any changes in eNOS expression or activity. Similar beneficial effects by empagliflozin are noted when CMECs are incubated in the presence of TNFα and interleukin-1β (IL-1β) [73]. Murine aortic rings cultured in hyperglycemic conditions display severely blunted endothelial NO-dependent vasodilation that is dependent on ROS production and corrected by empagliflozin [74]. The effect of empagliflozin appears class-specific as it is reproduced by dapagliflozin, and canagliflozin. Despite the restoration of NO and ROS levels, empagliflozin does not attenuate the TNFα-induced upregulation of adhesion molecules and EC barrier permeability [71]. Interestingly, a recent report found that empagliflozin, dapagliflozin, and canagliflozin ameliorate EC barrier dysfunction evoked by cyclic stretch through inhibition of ROS production, likely via blockade of the sodium-hydrogen exchanger 1 (NHE1) and NADPH oxidases (NOXs) [75]. Thus, the ability of SGLT2 inhibitors to prevent EC barrier dysfunction may be stimulus-dependent. 

### 3.2. SGLT2 Inhibitors Block EC Inflammation

SGLT2 inhibitors also elicits direct anti-inflammatory effects on ECs. In particular, incubation of cultured human ECs with clinically relevant concentrations of canagliflozin inhibits the IL-1β-stimulated adhesion of pro-monocytic U937 cells and the expression and secretion of interleukin-6 (IL-6) and monocyte chemoattractant protein-1 (MCP-1), whereas therapeutic levels of empagliflozin or dapagliflozin have no effect [76]. The anti-inflammatory effects of canagliflozin are not associated with any change in NF-κB signaling or adhesion receptor expression, but are paralleled by an increase in AMPK activity. Moreover, expression of a dominant-negative mutant of AMPK attenuates the inhibition of MCP-1 expression by canagliflozin, while the direct AMPK activator A769662 mimics the actions of canagliflozin, indicating that AMPK plays a fundamental role in mediating the anti-inflammatory actions of canagliflozin. In addition, canagliflozin blocks the release of IL-6 by lipopolysaccharide-stimulated ECs [77]. This anti-inflammatory effect of canagliflozin involves activation of AMPK activity and reduced hexokinase II expression. Administration of dapagliflozin also suppresses the hyperglycemia-mediated increase in intercellular adhesion molecule-1 (ICAM-1) expression, while empagliflozin decreases the adhesion of human mononuclear leukocytes to human ECs as well as the production of the chemokine C-C motif ligand 2 and 5 by angiotensin II [67,78]. Therefore, SGLT2 inhibitors afford protection against multiple inflammatory stimuli. 

### 3.3. SGLT2 Inhibitors Preserve the EC Glycocalyx

SGLT2 inhibitors may also limit inflammation by preserving the structural integrity of the endothelial glycocalyx. The glycocalyx is a mesh of proteoglycans, glycosamines, and glycolipids that lines the luminal surface of the endothelium. It serves as a barrier that protects the vessel wall from circulating inflammatory cells, limits vascular permeability, and acts as a sensor of mechanical forces, such as shear stress. Notably, the endothelial glycocalyx senses biomechanical stimuli triggering a host of intracellular events that lead to changes in endothelial morphology, the activation of eNOS, and release of NO [79]. Degradation of the glycocalyx by heparanase III abolishes mechanoactivation-induced NO synthesis by ECs and flow-induced vasodilation of resistance arteries. Acute and long-term hyperglycemia results in the degradation of the glycocalyx, leading to increases in vascular permeability, impaired mechanotransduction, and endothelial dysfunction in both diabetic patients and mice [80,81,82]. Interestingly, empagliflozin is able to restore the glycocalyx of heparinase III-treated human abdominal ECs by attenuating the degradation of heparin sulfate and possibly by re-establishing the heparin sulfate moiety [83]. Importantly, empagliflozin rescues the cellular elongation response to fluid flow in heparinase III-conditioned ECs and also blunts the interaction between ECs and neutrophil-like NB4 cells. Collectively, these findings indicate that empagliflozin reinstates mechanotransduction and the anti-inflammatory phenotype in ECs by maintaining the glycocalyx. In addition, a recent clinical study reported that 12-month treatment of T2DM patients with glucagon-like peptide-1 receptor agonists and SGLT2 inhibitors results in a significantly thicker endothelial glycocalyx than that seen in patients treated with insulin [84]. Moreover, the increase in glycocalyx thickeness by dual therapy correlates with improved arterial function, highlighting the potential importance of targeting the glycocalyx in diabetes. 

### 3.4. SGLT2 Inhibitors Regulate Vascular Repair and Angiogenesis

More recently, we discovered that SGLT2 inhibitors block the angiogenic response of human and murine ECs [85]. Treatment of HUVECs with clinically relevant concentrations of canagliflozin inhibits cell proliferation, while supra-pharmacological levels of empagliflozin and dapagliflozin are needed to block EC growth. Canagliflozin curbs the growth of human and mouse aortic ECs in a concentration-dependent fashion, but the degree of inhibition is greater in human relative to mouse cells, indicating that human cells are more sensitive to the anti-proliferative action of canagliflozin. These results are in contrast with other reports showing that canagliflozin has no effect on EC proliferation [76,86]. Discrepancies between these studies may reflect differences in treatment duration, where the utilization of longer duration experiments may better detect the anti-proliferative nature of canagliflozin and more accurately mimic the chronic administration of the drug in human patients. The blockade of EC growth by canagliflozin occurs in the absence of cell death and is associated with a reduction in DNA synthesis, the arrest of ECs in the G_0_/G_1_ phase of the cell cycle, and a profound decline in cyclin A expression. Although SGLT2 is expressed in HUVEC [76,85], it is unlikely to mediate the anti-proliferative action of canagliflozin, since similar effects would have been noted with pharmacologically relevant concentrations of empagliflozin or dapagliflozin. Instead, we found that restoration of cyclin A levels rescues the proliferative response of canagliflozin-treated ECs, establishing cyclin A as a critical new target of canagliflozin. The mechanism underlying the inhibition of cyclin A expression by canagliflozin is not known, but may be related to the blockade of mitochondrial metabolism. Recent reports indicate that the metabolism of glutamine by the tricarboxylic acid (TCA) cycle plays a central role in stimulating cyclin A expression and EC growth [87,88,89]; however, canagliflozin blocks mitochondrial glutamate dehydrogenase and complex I activity, preventing the replenishment of TCA cycle intermediates by glutamine which are needed for cell proliferation [90]. 

Canagliflozin also modestly inhibits the migration of ECs and markedly attenuates the differentiation of ECs into tubes and the sprouting of capillaries from mouse aortas [85]. The ability of canagliflozin to robustly inhibit EC proliferation and tube formation is of potential pharmacological importance as these cellular processes are involved in vascular repair and angiogenesis. In fact, canagliflozin was recently shown to limit intra-tumor vascularization in a xenograft model of liver cancer and may be of use in patients with proliferative diabetic retinopathy, where uncontrolled EC proliferation and angiogenesis initiates the disease [86,91]. Of concern, canagliflozin may further compromise limb blood flow in T2DM patients with peripheral artery disease by blocking angiogenesis. This may, in part, explain the significant risk of lower limb amputation by canagliflozin in this patient population, which is not seen with other SGLT2 inhibitors [37,38,39,40]. Consistent with this hypothesis, oral administration of canagliflozin for 8 weeks impedes the recovery of hindlimb function following unilateral hindlimb ischemia in diabetic db/db mice [92]. The canagliflozin-treated diabetic mice exhibit smaller gastrocnemius muscle fiber sizes, decreased microvascular density, and diminished perfusion compared to diabetic mice feed a control diet. Notably, canagliflozin also impairs hindlimb function and perfusion in wild-type mice undergoing unilateral hindlimb ischemia, suggesting that the actions of canagliflozin are not dependent on a hyperglycemic environment. Similarly, canagliflozin hampers angiogenesis and blood reperfusion in the ischemic lower limb of diabetic mice fed a high fat diet [93]. The impairment in hindlimb perfusion by canagliflozin is partially mediated by blocking the retention and paracrine function of bone marrow-derived mesenchymal stem cells (BM-MSCs). In particular, canagliflozin inhibits the proliferation and migration, and increases the apoptosis of BM-MSCs. These effects by canagliflozin likely occur due to mitochondrial dysfunction as evidenced by reductions in ATP production, oxygen consumption, and glutamate dehydrogenase activity. BM-MSCs paracrine function, as reflected by reduced exosome and vascular endothelial growth factor (VEGF) secretion, is also compromised by canagliflozin. Moreover, the therapeutic efficacy of BM-MSC transplantation in restoring hindlimb perfusion is compromised when BM-MSCs are preconditioned with canagliflozin. Surprisingly, an earlier study found that canagliflozin accelerates the recovery of hindlimb blood flow following femoral artery ligation and excision in diabetic NOD/SCID mice [94]. Furthermore, dapagliflozin was shown to promote neovascularization in the ischemic hindlimb of STZ-treated mice fed a high fat diet by improving paracrine function of skeletal muscle cells through the prolyl hydroxylase domain 2/hypoxia-inducible factor -1α signaling axis [95]. The reason for these conflicting reports is not entirely clear but may be related to the use of different SGLT2 inhibitors, the dose and duration of SGLT2 inhibitor treatment, and/or utilization of alternative animal models of diabetes. 

The mobilization of circulating stem cells (CSCs) and endothelial progenitor cells (EPGs) also promotes angiogenesis and vascular repair following injury. However, treatment of T2DM patients with dapagliflozin or empagliflozin for 12 weeks does not significantly increase CSCs or EPGs in these patients [96]. Similarly, a pilot study found that short-term treatment with canagliflozin fails to elevate EPGs in diabetic individuals, but did improve EPG migratory function in a manner that paralleled a rise in eNOS expression, suggesting a possible involvement of EPGs in the repair process [97]. Intriguingly, a recent report found that dapagliflozin enhances endothelial repair of injured carotid arteries in STZ-treated diabetic mice by restoring the mobilization and trafficking of CD49d+ granulocytes to sites of vascular damage [98]. The ability of SGLT2 inhibitors to facilitate the trafficking of bone marrow-derived hematopoietic cells to areas of arterial damage, illustrates a novel aspect by which SGLT2 inhibitors provide vascular protection. 

### 3.5. SGLT2 Inhibitors Suppress EC Senescence 

Recently, EC senescence has been identified as a potential mediator of endothelial dysfunction in diabetes. This process is characterized by irreversible growth arrest, activation of tumor suppressor genes, inflammation, oxidative stress, apoptosis, and downregulation of eNOS-derived NO formation [99]. Senescent ECs have been detected in the aorta of diabetic rats, and ECs subjected to high glucose concentrations exhibit increases in senescence-associated β-galactosidase activity [100,101,102]. In porcine ECs, high glucose-induced senescence results in a decline in eNOS expression and NO synthesis, a rise in oxidative stress and inflammation, and a local activation of the angiotensin system [103]. However, empagliflozin and the dual SGLT1/2 inhibitor LX-4211 prevents the induction of senescence and the phenotypic changes in ECs, and these effects are associated with an upregulation of SGLT1/2 receptors. Both drugs also reduce glucose uptake in the presence of high levels of glucose, suggesting that SGLT2 inhibitors may attenuate endothelial glucotoxicity in diabetes. In agreement with these results, CMECs isolated from diabetic mice have higher rates of senescence compared to cells attained from non-diabetic control animals [104]. Mechanistic studies revealed that empagliflozin inhibits CMEC senescence via an AMPK-mediated suppression of mitochondrial fission and a subsequent drop in mitochondrial ROS generation. By preventing cellular senescence through neutralizing fission-induced ROS production, empagliflozin also improves CMEC permeability and barrier function, and this is paired with a suppression of ICAM-1 and VCAM-1 expression and restoration of eNOS activity. In addition, empagliflozin-mediated repression of mitochondrial fission restores the migratory capacity of diabetic CMECs by preserving F-actin homeostasis. Empagliflozin also represses the expression of senescence markers in atheroprone regions of the aorta in Zucker diabetic lean rats [65]. 

### 3.6. SGLT2 Inhibitors Promote EC Viability

SGLT2 inhibitors may also influence the viability of ECs. The culture of HUVECs in a hyperglycemic environment results in the loss of NO synthesis and an increase in cell death that is reversed by empagliflozin [64]. Empagliflozin also prevents the death of cultured human microvascular ECs exposed to hypoxia/reoxygenation conditions and increases the survival of murine coronary endothelium following cardiac ischemia-reperfusion injury [105]. In both instances, the protective effect of empagliflozin is dependent on the activation of signal transducer and activator of transcription 3 (STAT-3). However, the precise EC death pathways that are affected by empagliflozin requires additional exploration.

## 4. Expression of SGLT2 in Vascular Cells

Several studies have demonstrated the presence of SGLT2 in vascular endothelium. Expression of SGLT2 protein is detected in cultured HUVECs, human aortic ECs, and human coronary artery ECs, but SGLT2 mRNA is not observed in these cells or in human CMECs [70,71,72,76,78,85]. The receptors appear functional as SGLT2 inhibitors are capable of blocking glucose uptake by human ECs [76]. Both SGLT2 protein and mRNA are expressed by cultured porcine coronary artery ECs and a weak immunofluorescence signal for SGLT2 is detected in the luminal surface of porcine coronary artery segments [103]. Similarly, SGLT2 protein and mRNA is present in murine aortic blood vessels, and an operative SGLT2 receptor is detected in cultured mouse aortic ECs [74]. Thus, SGLT2 expression is observed across various blood vessels and animal species. In addition, SGLT2 expression is regulated in a dynamic manner in ECs by various biochemical stimuli, including palmitic acid, hydrogen peroxide, hyperglycemia, TNFα, and angiotensin II [70,71,85,103,106]. However, the signaling pathways responsible for the induction of SGLT2 in ECs have not been delineated. Finally, less is known regarding the expression and functional relevance of SGLT2 in vascular smooth muscle; but, SGLT2 protein and mRNA is detected in rat and human aortic VSMCs, and the expression is upregulated by interleukin-17A [107,108]. 

## 5. Effect of SGLT2 Inhibitors on VSMC Function and Arterial Remodeling

SGLT2 inhibitors have also been demonstrated to modify VSMC function. We recently discovered that clinically relevant concentrations of canagliflozin inhibits the proliferation and migration of human and rat aortic VSMCs [109]. These inhibitory actions of canagliflozin are concentration-dependent and occur in the absence of cell death. The anti-proliferative action of canagliflozin is associated with the arrest of VSMCs in the G_0_/G_1_ phase of the cell cycle and a prominent decrease in DNA synthesis. In addition, canagliflozin stimulates the transcription of the heme oxygenase-1 (HO-1) gene, leading to a rise in VSMC HO-1 activity. The induction of HO-1 by canagliflozin is dependent on the activation of the ROS-NF-E2-related factor-2 (Nrf2) signaling pathway, as it is prevented by antioxidants or by overexpressing dominant-negative Nrf2. In contrast, empagliflozin and dapagliflozin fails to upregulate HO-1 activity, suggesting that the induction of HO-1 represents a compound-specific effect rather than a class effect. Significantly, the induction of HO-1 contributes to the anti-proliferative and anti-migratory actions of canagliflozin. Silencing HO-1 expression increases the proliferation and migration of canagliflozin-challenged VSMCs, and the exogenous administration of the HO-1 products, carbon monoxide and bilirubin, can substitute for HO-1 and rescue the anti-proliferative and anti-migratory response of canagliflozin-treated cells. Collectively, these findings indicate that HO-1-derived carbon monoxide and bilirubin underlie the ability of canagliflozin to block VSMC growth and motility. However, knockdown of HO-1 expression does not completely restore VSMC function, suggesting that other factors may also participate in mediating the functional effects of canagliflozin. In this respect, we previously identified AMPK as a potent inhibitor of VSMC proliferation and migration, providing an additional pathway by which canagliflozin regulates VSMC function [110]. Therapeutic concentrations of empagliflozin have likewise been shown to inhibit proliferation and DNA synthesis in rat aortic VSMCs [107]. Interestingly, the attenuation of VSMC growth by empagliflozin is only seen when cells are grown in high, but not low, glucose medium, suggesting that the drug elicits its anti-proliferative action by blocking glucose uptake through SGLT2. More recently, empagliflozin has also been demonstrated to block the proliferation and migration of human aortic VSMCs grown in normal glucose conditions and exposed to interleukin-17A [108]. In these cells, silencing SGLT2 expression does not fully abrogate the effects of empagliflozin, indicating that some of the functional actions of the drug occur in a SGLT2-independent manner. 

SGLT2 inhibitors also inhibit the contraction of VSMCs. Dapagliflozin dilates pre-contracted rabbit aortic rings in a concentration-dependent manner [111]. The vasodilatory effect of dapagliflozin is independent of the endothelium and relies on the direct activation of protein kinase G and voltage-dependent potassium channels. Acute administration of dapagliflozin also relaxes isolated aortic rings from mice in a dose-dependent and endothelium-independent fashion [67]. Similarly, phlorizin and canagliflozin relaxes murine pulmonary arteries in a dose-dependent manner [112]. However, vasorelaxation induced by these SGLT2 inhibitors may be vascular bed-dependent since they have no effect on coronary arteries. Interestingly, chronic delivery of canagliflozin significantly enhances sodium nitroprusside-dependent relaxation in coronary, but not in pulmonary, arteries of diabetic mice. In addition, dapagliflozin completely restores the NO donor sodium nitroprusside-mediated vasodilation of mesenteric arteries in db/db mice [68]. These latter findings suggest that SGLT2 inhibitors differentially regulate vascular relaxation in diabetes depending on the type of arteries and duration of treatment. Whether SGLT2 inhibitors affect other properties of VSMCs or influences phenotypic switching in these cells remains to be investigated. 

Emerging evidence indicates that SGLT2 inhibitors improve vascular remodeling in various pathological states. An early report found that glycemic control with empagliflozin ameliorates pericoronary arterial fibrosis, coronary arterial thickening, and vasodilating dysfunction in db/db mice [113]. These beneficial effects of empagliflozin are associated with diminished oxidative stress in cardiovascular tissues. Subsequently, it was determined that empagliflozin tends to attenuate neointima formation following guidewire-induced endothelial denudation injury of femoral arteries in db/db mice, while the combined treatment with empagliflozin and linagliptin strikingly blocks intimal thickening after injury [107]. In addition, the SGLT2 inhibitor ipragliflozin suppresses cuff-induced neointima formation of femoral arteries of apoE-deficient mice, and the migration of VSMCs challenged with platelet-derived growth factor (PDGF)-B [114]. Furthermore, luseogliflozin limits neointimal hyperplasia after wire injury in mice fed a high fat diet [115]. The beneficial effect of luseogliflozin is likely mediated via the reduced infiltration of PDGF-B-expressing macrophages into perivascular adipose tissue. Given its anti-inflammatory effects, the restoration of adiponectin expression by luseogliflozin may contribute to the limitation of macrophage infiltration into perivascular adipose tissue [115]. Empagliflozin also reduces pulmonary vascular remodeling, right ventricular hypertrophy and fibrosis, and mortality in the monocrotaline rat model of pulmonary hypertension [116]. Empagliflozin-treated animals display less pulmonary artery muscularization and medial wall thickness relative to vehicle control rats and this is coupled with increased rates of apoptosis and reduced rates of proliferation within the pulmonary arterial wall. Moreover, empagliflozin retards exercise-induced pulmonary hypertension in obese ZSF1 rats [117]. 

Administration of empagliflozin also antagonizes angiotensin II-induced dissecting abdominal aortic aneurysms in apoE-null mice [118]. The protection afforded by empagliflozin is dose-dependent and is independent of the blood glucose- and blood pressure-lowering actions of the drug. It is associated with a decline in the expression of inflammatory chemokines, VEGF, matrix metalloproteinase (MMP)-2 and MMP-9, and reduced macrophage infiltration into the aortic walls of apoE-knockout mice. Empagliflozin-treatment also reduces the vascular activation of p38 mitogen-activated protein kinase (MAPK) and NF-κB, which have been implicated in the development of aortic aneurysms [118,119]. The impairment of NF-κB activation by empagliflozin is noteworthy as it would limit the release of inflammatory and proangiogenic molecules, and result in blunted vascular cell activation. Importantly, a recent clinical study demonstrated that empagliflozin attenuates neointimal hyperplasia after drug-eluting stent implantation in patients with T2DM [120]. In particular, patients given empagliflozin in addition to standard therapy for 12 months after coronary stenting had significantly less neointima compared to patients receiving intense therapy with other glucose-lowering drugs. Changes in blood pressure is the strongest predictor for neointimal hyperplasia reduction, whereas no correlation with changes in blood glucose parameters is observed. This work supports the use of SGLT2 inhibitors in diabetic patients undergoing coronary revascularization therapy. 

## 6. Effect of SGLT2 Inhibitors on Arterial Stiffness

Arterial stiffening is a major contributor and predictor of cardiovascular disease and mortality [121]. Development of arterial stiffness is an intricate process that involves vascular cell proliferation, migration, hypertrophy, and alterations in the composition of the extracellular matrix [122,123]. These changes largely occur in the media and intima of blood vessels, and are primarily characterized by the fragmentation and disappearance of elastin with a corresponding increase in collagen and calcium deposition. While arterial stiffness increases with ageing, this process is accelerated and observed earlier in the presence of obesity, insulin-resistance, and diabetes [124,125]. Moreover, prevailing evidence shows that arterial stiffness is not only a central element in the pathogenesis of T2DM but it also has an independent predictive value for vascular complications associated with the disease [126]. Thus, interventions aimed at attenuating arterial stiffness are being developed to improve cardiovascular disease outcomes in patients with obesity, diabetes, and insulin-resistance [127,128]. 

Multiple studies indicate that empagliflozin dampens arterial stiffening in diabetes. A post hoc analysis of data from five clinical trials revealed that empagliflozin reduces pulse pressure, a surrogate measure of arterial stiffness, in patients with T2DM [129]. Sub analysis of a clinical trial comprising 58 patients with T2DM reported that therapy with empagliflozin improves arterial stiffness as indicated by reduced central systolic blood pressure, central pulse pressure, and reflected wave amplitude compared to placebo [130]. Aside from age and sex, changes in systolic 24-h ambulatory blood pressure and high sensitivity C-reactive protein are determinants of the empagliflozin-induced improvement in arterial stiffness, linking an anti-inflammatory effect to the salutary vascular effects of the drug. Empagliflozin is also associated with a decline in arterial stiffness in young T1DM subjects [131]. Furthermore, a preclinical study found that empagliflozin reduces systemic and renal artery stiffness in db/db mice [61]. Dapagliflozin also improves arterial stiffness in diabetic animals, while a pilot study found that it eases aortic stiffness in T2DM patients [68,132]. However, a larger follow-up trial failed to detect any effect of the drug on arterial stiffness in diabetic patients [59]. Post hoc analysis of five clinical trials showed that canagliflozin improves pulse pressure in patients with T2DM [133]. More recently, tofogliflozin was demonstrated to attenuate arterial stiffness, as reflected by a decrease in brachial-ankle pulse wave velocity, in patients with T2DM, whereas luseogliflozin fails to improve arterial stiffness in this patient population, as assessed by cardio-ankle vascular index and pulse wave velocity [134,135]. Failure of luseogliflozin to mitigate arterial stiffening may reflect the limited sample size and study duration. The mechanism by which SGLT2 inhibitors attenuate arterial stiffness is not fully known, but may be related to the suppression of AGE expression, decreased nitrosative stress, increased bioavailability of NO, attenuated accumulation of VSMCs within the vessel wall, and/or diminished arterial fibrosis [61]. 

## 7. Summary and Future Directions

**Figure 1:**
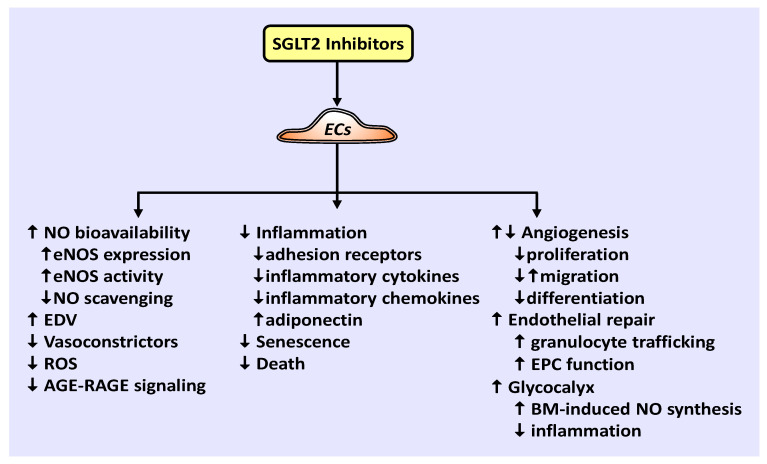
Effect of SGLT2 inhibitors on EC function. SGLT2 inhibitors increase NO bioavailability by elevating eNOS expression and activity, and/or limiting NO scavenging, thereby promoting endothelium-dependent vasodilation (EDV). In addition, SGLT2 inhibitors block the generation of vasoconstrictors and ROS, the activation of the AGE-RAGE signaling pathway, and EC death and senescence. They also attenuate inflammation by inhibiting the endothelial expression of adhesion receptors, inflammatory cytokines and chemokines, while enhancing the expression of adiponectin. Furthermore, SGLT2 inhibitors stimulate endothelial repair by improving EPC function and increasing the trafficking of granulocytes to injured arteries. SGLT2 inhibitors also preserve the structural integrity of the endothelial glycocalyx, resulting in sustained biomechanical (BM)-induced NO synthesis and a reduction in inflammation. Finally, SGLT2 inhibitors suppress EC proliferation and differentiation, but elicit variable effects on angiogenesis and EC migration.

There is a growing appreciation for the role of SGLT2 inhibitors in preventing cardiovascular disease and mortality via mechanisms beyond glucose control. Emerging work indicate that SGLT2 inhibitors play a crucial role in promoting vascular homeostasis by regulating vascular cell function and arterial remodeling. A model illustrating the effects of SGLT2 inhibitors on EC function is shown in Figure 1. Of particular importance, SGLT2 inhibitors have been demonstrated to improve the bioavailability of NO, which is intimately associated with vascular health. These drugs enhance NO levels by increasing the expression of eNOS, the phosphorylation and activation of eNOS, and/or decreasing the scavenging of NO by ROS. Through these actions, SGLT2 inhibitors have been documented to restore endothelium-dependent vasodilation in patients with T2DM and in animal models of diabetes. They also restrict the generation of vasoconstrictors to further vasodilation in diabetes. In addition, SGLT2 inhibitors exert many salutary effects on ECs. They inhibit the endothelial production of ROS, the activation of AGE-RAGE signaling, EC death and senescence, and stimulate endothelial repair at sites of arterial damage by improving EPC function and recruiting granulocytes to the region of injury. Furthermore, SGLT2 inhibitors are potent repressors of inflammation that prevent the endothelial expression of adhesion receptors, pro-inflammatory cytokines, and inflammatory chemokines, while elevating the expression of the anti-inflammatory adipokine, adiponectin. Moreover, SGLT2 inhibitors dampen inflammation and restore biomechanical signaling-induced NO-mediated vasodilation by augmenting the thickness of the endothelial glycocalyx. The effect of SGLT2 inhibitors on angiogenesis is unclear as both increases and decreases in angiogenesis have been reported. Similarly, while canagliflozin inhibits the proliferation, migration, and differentiation of cultured ECs into tubes under normoglycemic conditions, empagliflozin restores the migration of diabetic ECs. Clearly, additional studies are needed to precisely determine the effect of individual SGLT2 inhibitors on the angiogenic response of ECs under various experimental conditions. 

**Figure 2:**
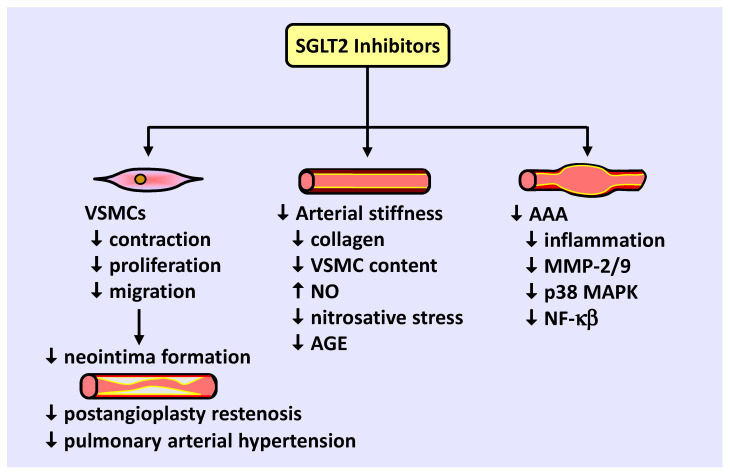
Effect of SGLT2 inhibitors on VSMC function and arterial remodeling. SGLT2 inhibitors block VSMC contraction, proliferation, and migration, leading to vasodilation and diminished neointima formation following coronary angioplasty or in pulmonary arterial hypertension. In addition, SGLT2 inhibitors attenuate arterial stiffening by depressing collagen deposition, VSMC accumulation within the vessel wall, nitrosative stress, and AGE expression, and raising NO levels. SGLT2 inhibitors also reduce abdominal aortic aneurysm formation by blocking vascular inflammation and MMP-2/9 activity, possibly through inhibition of p38 MAPK and NF-κB activation.

SGLT2 inhibitors also modulate VSMC function and arterial remodeling (Figure 2). Studies from our laboratory and others found that SGLT2 inhibitors block VSMC contraction, proliferation, and migration, leading to vasodilation and diminished neointima formation. Preclinical and clinical studies support a role for SGLT2 inhibitors in retarding blood vessel stenosis following arterial injury and alleviating the maladaptive remodeling of pulmonary arterioles in pulmonary arterial hypertension. SGLT2 inhibitors also reduce arterial stiffness in diabetic humans and animals by limiting collagen deposition and VSMC content within the vessel, potentially by increasing NO synthesis and reducing nitrosative stress and AGE expression. In addition, SGLT2 inhibition prevents the development of abdominal aortic aneurysm in mice by limiting inflammation and MMP-2/9 expression by targeting specific upstream signaling pathways. Collectively, these actions by SGLT2 inhibitors illustrate a novel promising therapeutic strategy to prevent occlusive and dissecting vascular disease.

Notably, the beneficial effects of SGLT2 inhibitors on vascular cell function and remodeling are observed in the presence and absence hyperglycemia, suggesting that SGLT2 inhibitors will be effective in treating vascular disease beyond the diabetic population. This is in-line with recent work showing dapagliflozin attenuates the risk of worsening cardiac dysfunction and cardiovascular death in patients with heart failure and reduced ejection fraction, regardless of the presence or absence of diabetes [43]. Similarly, a recently completed study found that empagliflozin lowers the risk of cardiovascular death and hospitalization for heart failure in both diabetic and non-diabetic patients [136]. Thus, both the vascular and cardiac protective actions of SGLT2 inhibitors are independent of their glucose-lowering effect. 

While substantial progress has been made in determining the effect of SGLT2 inhibitors on vascular cell function, important questions remain. Studies have detected the presence of SGLT2 in vascular cells and glucose entry in ECs via SGLT2; however, it remains unclear whether the beneficial effects of SGLT2 inhibitors occur via the inhibition of this transporter. Future gain- and loss-of-function studies targeting SGLT2 in vascular cells are needed in order to establish whether the cellular actions of these drugs are dependent on SGLT2. Similarly, vascular cell-specific SGLT2 knockout animals should be generated to test whether SGLT2 mediates the salutary effects of SGLT2 inhibitors on vascular remodeling. This is an important issue as there is a growing realization that SGLT2 inhibitors elicit numerous off-target effects. This is especially the case for canagliflozin. Clinically relevance concentrations of this drug have been reported to activate AMPK, stimulate HO-1 gene expression, repress cyclin A protein expression, block mitochondrial glutamate dehydrogenase and mitochondrial complex I activity, and inhibit the activity of uridine 5′-diphospho-glucuronlsyltransferase and the sodium-hydrogen exchanger [85,90,109,137,138,139,140]. In fact, the inhibition of endothelial inflammation by canagliflozin has been ascribed to the activation of AMPK, while the inhibition of VSMC proliferation and migration by this SGLT2 inhibitor is linked to the induction of HO-1 [76,109]. This underscores the need to consider these pleiotropic effects when assessing the effect of SGLT2 inhibitors on vascular cell function. Interestingly, SGLT2 inhibitors alter cellular metabolism by reducing glucose oxidation and increasing lipid oxidation resulting in a shift toward ketone production [141,142]. Given the importance of glycolysis and lipolysis in modulating vascular cell phenotype [143], examination of the capacity of SGLT2 inhibitors to rewire vascular cell metabolism represents an attractive area of investigation. A better understanding of the pleiotropic and metabolic actions of the various SGLT2 inhibitors may uncover extra potential benefits for specific SGLT2 inhibitors and aid in the selection of the optimal SGLT2 inhibitor for specific cardiovascular complications. 

In conclusion, SGLT2 inhibitors are well tolerated drugs that reduce the incidence of cardiovascular death and heart failure hospitalization in patients with and without diabetes. The mechanism by which SGLT2 inhibitors exert their beneficial effects is not fully known; however, their ability to regulate vascular cell proliferation, migration, differentiation, oxidative stress, inflammation, contractility, senescence, and death, and also mitigate aberrant arterial remodeling is of prime importance. Intriguingly, their pharmacological properties extend beyond SGLT2 inhibition and glycemic control, suggesting their potential use in a wide spectrum of cardiovascular disorders. Future studies that fully characterize the functional, metabolic, and molecular actions of individual SGLT2 inhibitors in vascular cells may provide additional insight into how these drugs prevent the development vascular disease and may lead to a more refined therapeutic approach that utilizes specific SGLT2 inhibitors in distinct patient populations.
